# Femtosecond Laser Ablated FBG with Composite Microstructure for Hydrogen Sensor Application

**DOI:** 10.3390/s16122040

**Published:** 2016-12-01

**Authors:** Meng Zou, Yutang Dai, Xian Zhou, Ke Dong, Minghong Yang

**Affiliations:** National Engineering Laboratory for Fiber Optic Sensing Technology, Wuhan University of Technology, Luoshi Road 122, Wuhan 430070, China; zoumeng209@163.com (M.Z.); chinazhoul@163.com (X.Z.); whutdongke@163.com (K.D.)

**Keywords:** fiber optics sensor, thin films, laser materials processing

## Abstract

A composite microstructure in fiber Bragg grating (FBG) with film deposition for hydrogen detection is presented. Through ablated to FBG cladding by a femtosecond laser, straight-trenches and spiral micro-pits are formed. A Pd–Ag film is sputtered on the surface of the laser processed FBG single mode fiber, and acts as hydrogen sensing transducer. The demonstrated experimental outcomes show that a composite structure produced the highest sensitivity of 26.3 pm/%H, nearly sevenfold more sensitive compared with original standard FBG. It offers great potential in engineering applications for its good structure stability and sensitivity.

## 1. Introduction

Hydrogen is an ideal gas for energy generation and has been widely used in many fields such as metallurgical processes, aerospace, and the biomedical industry [1]. However, as a colorless and tasteless flammable gas, hydrogen easily leaks without detection by human senses. As a result, it is essential to detect its concentration rapidly and accurately to avoid the disastrous consequences of hydrogen leakage [2,3].

In recent years different kinds of optical fiber hydrogen sensors have attracted more and more attention in industry, because of its advantages of anti-corrosion, anti-electric magnetic interference, and intrinsic safety [4]. Many optical fiber hydrogen sensors based on micro-mirror optical fiber sensors [5], fiber Bragg grating (FBG) sensors [6,7,8,9], interference sensors [10], and evanescent field sensors [11] have been investigated. Due to the advantages in wavelength division multiplex, self-reference, and anti-interference, FBG sensors are more suitable for monitoring hydrogen leaks for distribution measurement [12].

Due to the strong atomic bonding interaction with hydrogen gas, palladium (Pd) is a sensitive material used as a transducer layer between gas and optical waveguide [13,14,15]. Pd films can produce a physical and chemical reaction with hydrogen, and then the wavelength of FBG is changed by the mechanical stress of Pd film after exposure to hydrogen gas, but a pure Pd layer easily cracks and blisters, caused by its α–β phase transition [16]. Pd–Ag composite films have attracted wide attention because they can suppress the α–β transition to some extent and have good hydrogen permeating capacity [17]. However, the sensitivity is weak because the thickness of cladding layer is large for the transmission of stress. Therefore, improving the sensitivity would be a key issue for the development of a FBG hydrogen sensor. A number of researchers have reported chemical etching and side-polishing methods as a way to enhance the sensitivity [2,18,19], but the FBG becomes fragile after etching, and the thickness of side-polish is hard to control. Femtosecond lasers have high micromachining accuracy and small thermal deformation on the surface of materials [20,21,22]. Thus, laser processing has obvious superiority for the manufacture of microstructures on the FBG cladding.

In this paper, an optical fiber FBG hydrogen sensor integrated with composite microstructure and sensitive material is proposed. The straight-trenches and spiral micro-pits on FBG cladding fabricated by femtosecond laser micromachining are used to improve the sensitivity of the sensor. A Pd–Ag film to be used as the transducer layer between the gas and the optical fiber waveguide for monitoring hydrogen concentration is deposited on the composite microstructure by a magnetron sputtering process.

## 2. Principle of Sensor

**Figure 1:**
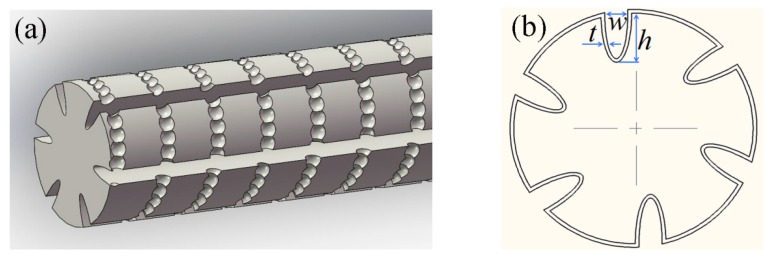
(**a**) Schematic profile diagram of the fiber Bragg grating (FBG) sensor; (**b**) simplified sectional view of the sensor.

A schematic profile diagram of the proposed FBG sensor is shown in Figure 1. The straight-trenches and spiral micro-pits were fabricated on FBG cladding by femtosecond laser micromachining, which can improve the sensitivity of the sensor, while the spiral micro-pits can make the film–fiber bond stronger.

The fiber Bragg grating acts as a spectral filter that allows part of the incident signal to be reflected, which is expressed by

(1) $$\lambda_{B}=2n_{eff}\Lambda$$

where *λ_B_* is the Bragg wavelength. Λ is the period of the grating, and *n_eff_* is the effective refractive index. Differentiating Equation (1) yields:

(2) $$\Delta \lambda_{B}=2\Lambda \Delta n_{eff}+2n_{eff}\Delta \Lambda$$

where Δ*λ_B_* is the Bragg wavelength’s shift, Δ*n_eff_* is the variation of the effective index of the fiber core, and ΔΛ is the period change of FBG. When the FBG is exposed to hydrogen gas, the grating period of the fiber is increased slightly due to expansion of the Pd layer. The influence of Δ*n_eff_* is much smaller than that of ΔΛ after sputtering, and ΔΛ will dominate the change of Δ*λ_B_*; hence, Δ*n_eff_* can be neglected. The shift of Bragg wavelength in Equation (2) can be expressed simply as:

(3) $$\Delta \lambda_{B}≅2n_{eff}\Delta \Lambda$$

Equation (3) can be used to predict the peak shift of FBG wavelength under strain. When the FBG is coated with Pd, the stress associated with the hydrogen absorption in the film can be measured by monitoring the reflection or transmission spectra of the FBG. As the hydrogen is absorbed by the Pd film, it expands because hydrogen absorption converts Pd to PdH_x_, which has a lower density and a larger volume. 

The simplified cross-sectional view of the sensor is shown in Figure 1b with spiral micro-pits (not shown in the diagram). Six trenches are machined to the cladding layer, and the deviation of trenches from the fiber core center is about 10 μm to avoid chirping. Then, a hydrogen sensitive film is coated on the surface of the machined optic fiber. The main parameters of the microstructure are: the width (*w*), the depth (*h*), the number (*n*) of the trenches, the thickness of film (*t*), and the depth of micro-pits (*v*). The cross-sectional area of the machined optic fiber (*A*_1_) can be approximately expressed by:

(4) $$A_{1}=\frac{\pi D^{2}}{4}-nwh-k_{1}v$$

where *D* is the diameter of cladding layer, and *k*_1_ is a coefficient related to the micro-pit structure. As a result, the cross-sectional area of the hydrogen sensing film (*A*_2_) can be described as:

(5) $$A_{2}=(\pi D+2nh+K_{2}v)t$$

where *k*_2_ is another coefficient related to the micro-pit structure. In theory, the strain and wavelength shift of FBG increases with increasing *A*_2_ and decreasing *A*_1_. From Equations (4) and (5), increasing the main parameters (*w*, *h*, *n*, *t*, *v*) will raise the sensitivity of the FBG. However, fiber mechanical strength will be weaken with the increase of parameters *w*, *h*, *n* and *v*, and the ability of hydrogen to permeate the film will decrease with the increase of parameter *t*. Therefore, it is crucial to balance the proper fiber mechanical strength with higher sensitivity during the design of parameters.

## 3. Experiment

**Figure 2:**
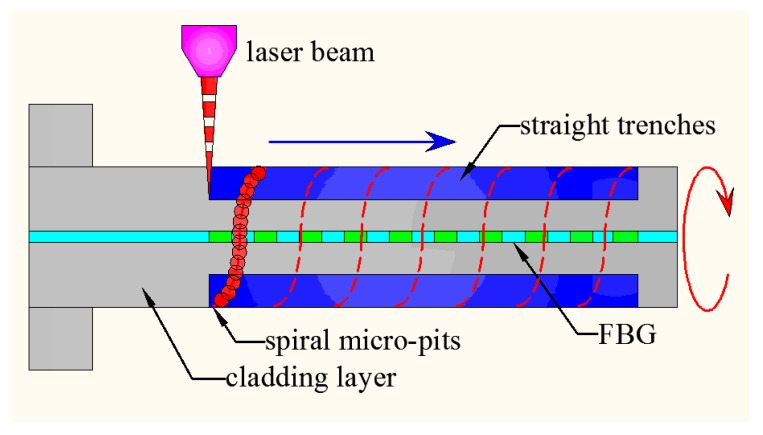
Schematic diagram for composite microstructure ablated by femtosecond laser.

**Figure 3:**
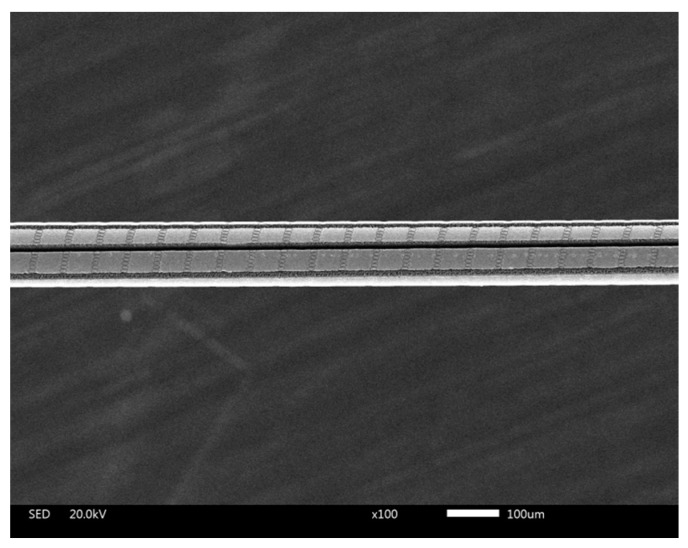
SEM figure of Pd/Ag thin film deposited onto composite structure.

A schematic diagram for the fiber composite microstructure ablated by femtosecond laser is shown in Figure 2. The operating wavelength of the femtosecond laser system (IFRIT-Cyber Laser) was about 780 nm, and the pulse width was about 180 fs. For the micromachining, a standard single-mode optical fiber (about 1532 nm of central wavelength and 125 μm fiber with 10 μm diameter core region) was used. Before fabrication of the microstructure, the polyimide protective layer on FBG (about 125 μm for a single side) was peeled off, and then FBG was fitted in a rotary fixture to fabricate 3D microstructures. Straight trenches are formed when the focused laser beam moves along the fiber surface as shown with the arrow in Figure 2. On the other hand, the machining of spiral micro-pits relies on the rotation of the fiber held by the jig fixture. A scanning electron microscope (SEM) image of the laser-induced composite structure coated with Pd/Ag film is shown in Figure 3.

As shown in Figure 1a, two types of microstructures are manufactured on the fiber cladding beneath the FBG by choosing laser parameters appropriately. Table 1 shows the main parameters of the two structures.

The attenuator can control the laser energy by changing the mode (Modes 1 and 2). Mode 1 means all energy (100%) passes through the attenuator, and Mode 2 means only 11% of energy passes through the attenuator. The aperture is used to change the size of the light spot. When the trench is ablated, the frequency is set to the maximum (1 KHz) in order to get a continuous groove. Similarly, in order to get interrupted micro-pits, the frequency is decreased to 10 Hz. The distance between two successive micro-pits (*L*) is ~10 μm, which depends on the frequency and sweep speed. Eventually, straight trenches (*n* = 4, 6, and 8) are incorporated to form composite microstructures. Figure 1b shows a schematic profile diagram with six straight trenches (*n* = 6).

According to the experience, some representative parameters are selected. Table 2 shows the laser machining parameters of the sensor samples. During spiral micro-pit micromachining, the laser energy and frequency are fixed at 440 mW and 10 Hz, respectively. This low frequency induces a minimal micro-pit depth on the fiber surface. The pitch of the spiral micro-pit samples (4#, 7#, 8#) are formed by adjusting rotation speed, while the laser energy of samples (4#, 9#, 10#) and numbers of the trench of samples (4#, 5#, 6#) increases the sensor sensitivity.

**Figure 4:**
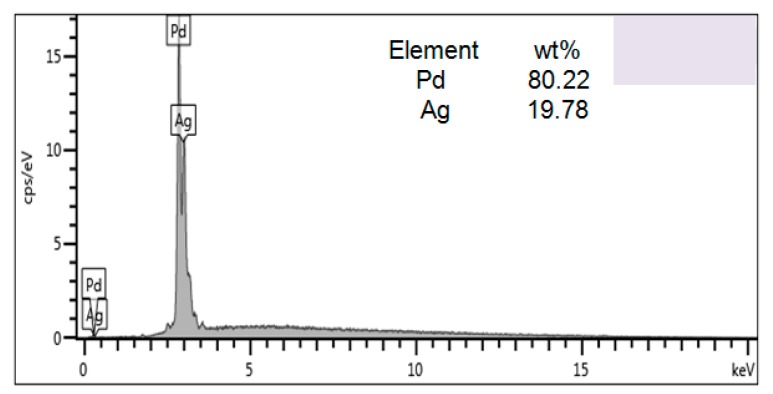
Spectral diagram of film elements.

The deposition of the hydrogen sensing film is conducted in a BESTECH sputtering system. Prior to thin film application, the machined fiber samples were ultrasonically cleaned with diluted HF for 2 min to remove fiber debris. Pd and Ag targets were installed to the DC source, and the pre-treated samples were located at bottom center. A thin Pd–Ag film was deposited on the surface of the FBG in high vacuum. Deposition power for palladium alloy targets is 100 W, which corresponds to a deposition rate of about 0.1 nm/s. The film thickness is controlled accurately using a crystal oscillator. In this experiment, about a film of about 500 nm thickness was deposited on the cladding surface of the micro-structured samples with the atomic ratio of Pd:Ag being 4:1, respectively, as shown in Figure 4. In order to ensure the uniformity of the coated film, the fiber rotation should be made continuous. However, dynamic rotation affects the deposition of the Pd layer and reduces the bonding strength of the Pd layer with fiber. Therefore, we selected a static deposition method during the sputtering process; i.e., after a two-hour depositing step completed, the samples were turned 180 degrees, then another two-hour depositing step was carried out.

**Figure 5:**
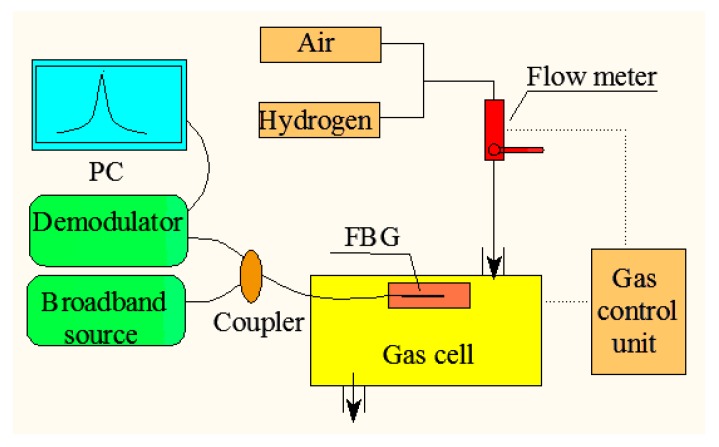
Experimental setup of hydrogen gas measurement.

The experimental setup for hydrogen gas measurement and characterization is shown in Figure 5. The hydrogen sensing performance was carried out in ambient air environment and at a room temperature of ~25 °C. One end of the FBG was fitted in a gas cell, and another end of the FBG was connected to a broadband source through a demodulator by a 3 dB coupler. The resolution of the demodulator was 0.1 pm. The varying hydrogen concentrations were monitored by the gas control unit. A real-time drifting FBG spectrum is observed in the computer.

## 4. Results and Discussion

**Figure 6:**
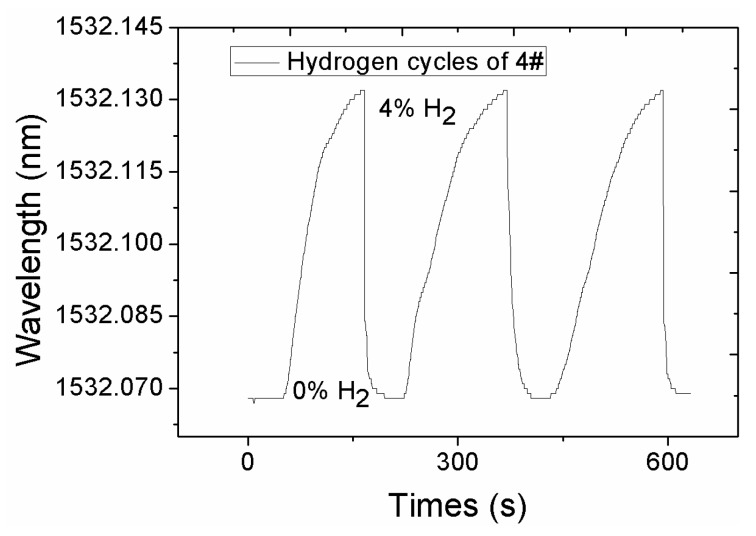
Response curve of composite-structured FBG to hydrogen.

The explosive limit of hydrogen in the air is about 4%–75%, so an initial threshold of 4% can be defined as a reference end-point. Figure 6 demonstrates a response curve of sensor sample 4# to hydrogen for three cycles from 0% to 4%. The average response time is about 140 s, which is superior to the pure Pd coating by several minutes. A pure Pd film will react and cause a phase transition which cannot be reversed to its original state. A Pd–Ag film reduces the response time effectively, as it can reduce the α–β phase shift change. The presence of Ag will accelerate the release of hydrogen. From Figure 6, it is also found that the sensor has good repeatability in varying hydrogen concentrations.

**Figure 7:**
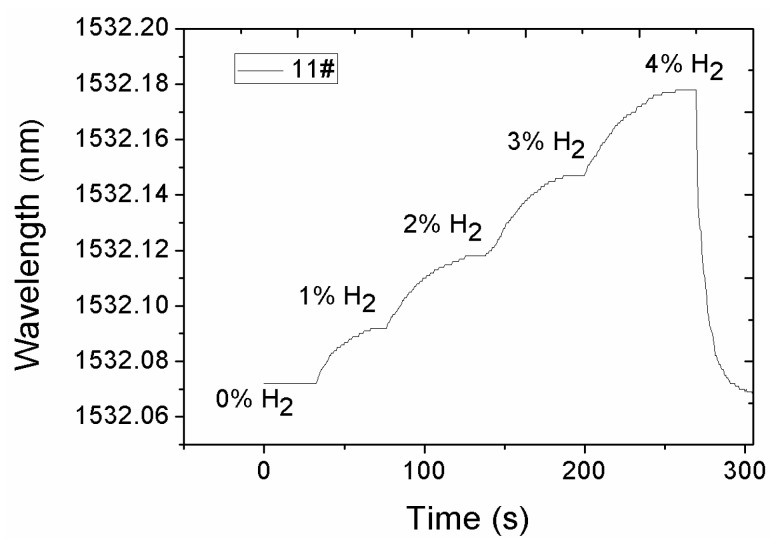
Response curve in different hydrogen concentrations.

Among all of the sensors shown in Table 2, sensor sample 11# with composite structure produced the highest sensitivity, as shown in Figure 7. When hydrogen concentrations are 1%, 2%, 3%, and 4%, the corresponding wavelength shifts of FBG are 20, 46, 75, and 105 pm, respectively. The sensitivity of the 11# sensor was estimated to be 26.3 pm/%H (wavelength shift caused by the change of 1% hydrogen concentration) through linear fitting. At first, the process of hydrogen absorption takes a long time as hydrogen diffuses through the thick Pd/Ag layer before volume expansion is realized. Thus, the wavelength shift is relatively small. As the hydrogen gas concentration increases and permeates the Pd/Ag film, a good response is realized.

**Figure 8:**
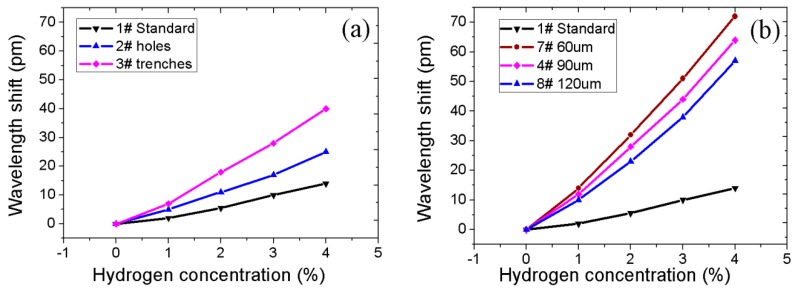
(**a**) Wavelength shift of sensors with single-structure; (**b**) Wavelength shift of sensors with different pitches.

Figure 8a shows the wavelength shift of sensors with a single structure—namely, trenches, micro pits, and standard FBG (without microstructure)—with their corresponding wavelength shifts at 4% being 40 pm, 25 pm, and 12 pm, respectively. Apparently, spiral micro-pits and trenches can individually improve the sensitivity of hydrogen sensors more than the unprocessed standard FBG sample. This can be explained by the fact that trenches greatly etch the cladding structure, thus increasing the surface area of the Pd/Ag composite layer contained in the trenches. On the other hand, the strain due to film expansion leads to FBG stretching within the fiber core. Spiral micro-pits benefit the deposition of the sensitive film as a cellular structure, thus, making the film–fiber bond stronger.

The effect of varying spiral micro-pits pitches on the wavelength shift due to the change in H_2_ concentration is presented in Figure 8b. Except the pitches, samples 4#, 7#, and 8# have the same machining parameters. It can be seen that the hydrogen gas sensitivity was highest in sample 7#, followed by 4# and 8#, respectively. The smaller the pitch of the spiral micro-pits, the higher the sensitivity to varying hydrogen concentrations. This can be attributed to the increased hydrogen-sensitive film surface area associated with smaller pitches as opposed to long pitches.

**Figure 9:**
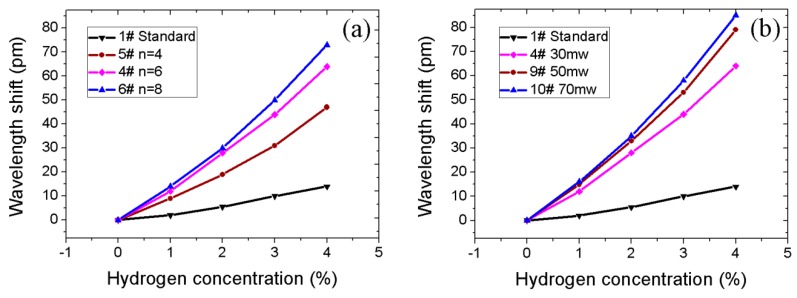
(**a**) Wavelength shift of sensors with different number of trenches; (**b**) Wavelength shift of sensors with different power.

Figure 9a illustrates the response of sensors with different numbers of trenches under different hydrogen concentrations. The wavelength shifts of samples 4#, 5#, and 6# were 47 pm, 64 pm, and 73 pm, respectively at 4%. It can be found that the sensitivity of the sensors increases with increasing number of trenches (*n*). However, the effect of spiral micro-pits compromises the increase of *n*. Theoretically, the maximum of *n* is about 12 under the restriction of fiber cross-section area. In order to optimize the fiber’s mechanical strength and hydrogen sensitivity of the two structures, *n* should be six or eight.

The influence of laser machining power on sensors’ sensitivity is shown in Figure 9b. The wavelength shifts of samples 4#, 9#, and 10# were 16, 20, and 21 pm/%H, respectively. The increase in laser energy is paramount, since it improves the sensors’ sensitivity by raising trench depth. When the energy was raised to 70 mw from 50 mw, the wavelength shift only improved by 6 pm. The reason behind this is that the relationship between trench depth and laser energy is non-linear. Since the femtosecond laser emits Gaussian beams, their characteristic limits the machining to form narrow and deep trenches, thus increasing the machined trench depth as laser energy increases. Taking into account various laser fiber parameter requirements for trench fabrication, suitable energy for machining is ~50 mw.

## 5. Conclusions

A composite microstructure sensor with straight-trenches and spiral micro-pits fabricated by a 780 nm femtosecond laser in the cladding of FBG has been demonstrated. A 500 nm-thick Pd–Ag film was sputtered on FBG cladding to form a hydrogen sensing probe. The composite structure has higher sensitivity and faster response time than that of single microstructure. For this reason, the proposed hydrogen sensor is a good candidate for detection. The composite structure comprised of straight trenches (*n* = 8) with 70 mw laser energy and spiral micro-pits with pitches (*p* = 60 μm) have the strongest response to hydrogen gas. Due to its high sensitivity and stable structure, the composite microstructure offers great potential in engineering applications for hydrogen detection.

## Figures and Tables

**Table 1:** The main parameters of two micromachining type.

Structural Type	Attenuator	Aperture	Frequency
trenches	Mode 2	No. 7	1000 Hz
micro-pits	Mode 1	No. 4	10 Hz

**Table 2:** The sample parameters.

Sample No.	Spiral Pitch (μm)	Trench Laser Energy (mW)	Number of Trenches (n)
1#	0	0	0
2#	90	0	0
3#	0	30	6
4#	90	30	6
5#	90	30	4
6#	90	30	8
7#	60	30	6
8#	120	30	6
9#	90	50	6
10#	90	70	6
11#	60	70	8
